# A Lightweight Human Fall Detection Network

**DOI:** 10.3390/s23229069

**Published:** 2023-11-09

**Authors:** Xi Kan, Shenghao Zhu, Yonghong Zhang, Chengshan Qian

**Affiliations:** 1 School of the Internet of Things Engineering, Wuxi University, Wuxi 214105, China; kanxi@cwxu.edu.cn (X.K.); qianchengshan@163.com (C.Q.); 2 School of Automation, Nanjing University of Information Science & Technology, Nanjing 211800, China; 20211249602@nuist.edu.cn

**Keywords:** YOLOv5, fall detection, GSConv module, GDCN module, NAM, SIoU

## Abstract

The rising issue of an aging population has intensified the focus on the health concerns of the elderly. Among these concerns, falls have emerged as a predominant health threat for this demographic. The YOLOv5 family represents the forefront of techniques for human fall detection. However, this algorithm, although advanced, grapples with issues such as computational demands, challenges in hardware integration, and vulnerability to occlusions in the designated target group. To address these limitations, we introduce a pioneering lightweight approach named CGNS-YOLO for human fall detection. Our method incorporates both the GSConv module and the GDCN module to reconfigure the neck network of YOLOv5s. The objective behind this modification is to diminish the model size, curtail floating-point computations during feature channel fusion, and bolster feature extraction efficacy, thereby enhancing hardware adaptability. We also integrate a normalization-based attention module (NAM) into the framework, which concentrates on salient fall-related data and deemphasizes less pertinent information. This strategic refinement augments the algorithm’s precision. By embedding the SCYLLA Intersection over Union (SIoU) loss function, our model benefits from faster convergence and heightened detection precision. We evaluated our model using the Multicam dataset and the Le2i Fall Detection dataset. Our findings indicate a 1.2% enhancement in detection accuracy compared with the conventional YOLOv5s framework. Notably, our model realized a 20.3% decrease in parameter tally and a 29.6% drop in floating-point operations. A comprehensive instance analysis and comparative assessments underscore the method’s superiority and efficacy.

## 1. Introduction

Elderly falls stand out as a primary contributor to injuries and fatalities, highlighting the gravity of this issue. The World Health Organization [1] emphasizes that falls are a significant cause of injuries, loss of autonomy, and hospital admissions for those aged 64 and above. The data suggest that individuals with fall-induced complications that lead to disability encounter a 50% elevated risk of mortality within the subsequent six months. Predominant age-associated factors, encompassing diminished physical functionalities like balance, vision, and coordination, and health ailments such as dizziness, muscular weakness, and arthritis, play a pivotal role in fall incidents. A lackluster diet, physical inertia, and sedentary behaviors further amplify the fall risk. A paramount challenge lies in devising a scientific approach to detect falls, promptly eliciting alerts for aid, all the while maintaining the elderly person’s routine activities unhampered. Instituting an astute elderly care system combined with an efficient fall detection mechanism is indispensable for societal advancement. Therefore, the surge in deep-learning-driven object detection techniques has propelled pedestrian fall detection research into the spotlight. Currently, deep-learning-driven object detection algorithms bifurcate into two categories: two-stage and one-stage methodologies. The two-stage paradigm encompasses algorithms like Faster R-CNN [2], R-FCN [3], Mask R-CNN [4], and Cascade R-CNN [5]. These strategies deploy a region proposal network to sketch object position propositions, which are subsequently classified and fine-tuned by a detached object detection network based on bounding box coordinates, culminating in the final detection verdict. Although two-stage algorithms commendably uphold precision, their speed often lags. In contrast, one-stage strategies, such as the YOLO series [6,7,8,9] and SSD [10], segment the image into grids, forecasting the object category and bounding box coordinates for each grid segment. The conclusive detection is then ascertained through post-processing. One-stage algorithms are lauded for their briskness and commendable real-time capabilities, albeit sometimes compromising on precision in comparison with their two-stage counterparts. Nevertheless, recent innovations in one-stage algorithms have considerably uplifted their accuracy, positioning them as a favored choice for a myriad of pragmatic applications.

Before the introduction of the YOLOv4 and YOLOv5 algorithms, YOLOv3 was extensively applied in fall-related tasks. Feng et al. [11] enhanced the YOLOv3-Tiny architecture by integrating four convolutional layers with 3 × 3 kernel dimensions. Although these modifications augmented the feature extraction capability and precision of detections, they concomitantly diminished the detection velocity. He et al. [12] proposed a lightweight network model tailored for fall detection, rooted in the YOLOv3 structure. This model incorporated the MobileNetV3 backbone network in conjunction with an SE [13] module. This innovative design astutely resolved challenges associated with an extensive model size and protracted inference speed, achieving a more streamlined model by markedly curtailing computational demands. Nonetheless, such optimization toward lightness inevitably curtailed the detection precision to some degree.

Introduced by Ultralytics in 2020, YOLOv5 is the latest iteration in the YOLO series at the time of our research. Like its predecessor YOLOv4, YOLOv5 employs CSPDarknet53 as its backbone network. The neck network of YOLOv5 incorporates the architecture of Feature Pyramid Networks (FPNs) [14] and a Pixel Aggregation Network (PAN) [15], essentially extending the concepts of feature pyramid and pixel aggregation.

YOLOv5, boasting significant enhancements in detection accuracy, speed, and model size, is widely adopted for object detection tasks across diverse fields [16,17,18]. Chen T et al. [19] advanced YOLOv5’s performance by substituting its backbone network’s basic convolutions with ACB convolutional blocks. This modification notably augmented the feature extraction ability. Nevertheless, such enhancement can increase computational complexity and memory demands. Peng et al. [20] fortified the feature extraction and recognition precision of YOLOv5s by integrating an Efficient Channel Attention (ECA) module and a Bi-directional Feature Pyramid Network (BiFPN) into the neck network. Despite its effectiveness, this strategy added complexity and parameters, elongating training and inference durations and elevating memory needs. Chen’s team [21] refined the YOLOv5s structure by discarding particular convolutional layers and incorporating Squeeze-and-Excitation Networks (SENets) channel attention modules. Although removing convolutional layers balances accuracy and speed, this could disturb receptive fields and local feature extraction capabilities, potentially undermining detection efficacy. Aiming to expedite inference speed, Chen et al. [22] merged convolutional and BN layers. This might, however, decelerate model convergence, introduce instability, and sacrifice feature information, possibly impairing performance and accuracy. Fu et al. [23] introduced a fall detection algorithm leveraging YOLOv5s and a lightweight OpenPose model, achieving a notable recognition accuracy. However, the necessary preprocessing of human body information entails intricate computational tasks. Nguyen HC and his team [24] combined YOLOv5 with High-Resolution Net (HRNet) for automated human pose detection and estimation, harnessing YOLOv5’s swift detection and HRNet’s prowess in 2D keypoint estimation. Regrettably, the multi-stage computation, encompassing detection and keypoint estimation, amplifies computational burden, lengthens training and inference phases, and diminishes real-time functionality. In addition to our research, several other studies have explored the application of lightweight neural networks in various domains. Li et al. [25] presented a lightweight convolutional neural network called WearNet, trained with optimized parameters including learning rate, gradient algorithm, and mini-batch size, which achieved an outstanding classification accuracy of 94.16% for automatic scratch detection in components subject to contact sliding, as seen in metal-forming processes. Wang et al. [26] primarily employed the YOLOv5s model, superpixel image segmentation, and the MobileNetv3 network to address interference from motion shadows in black smoke vehicle detection. It creatively adopts a “segmentation-classification” approach, enhancing accuracy and real-time performance. Zheng et al. [27] introduced an enhanced YOLOv5 algorithm for ship detection in surveillance videos. Key techniques include anchor box optimization based on ship characteristics and the use of the scaling factor to reduce the model size without sacrificing detection performance, resulting in increased accuracy and improved detection speed. Although YOLOv5 and HRNet contribute substantially to human fall detection, challenges persist. (1) A dilemma between accuracy and speed: several advanced algorithms prioritize detection accuracy and occlusion management, often resulting in numerous floating-point operations, sizable model files, and sluggish computational speeds, making them less ideal for real-time fall detection. (2) Inherent shortcomings of lightweight models: despite reducing parameters, many lightweight models grapple with optimizing both accuracy and speed, thus compromising their efficacy on resource-constrained edge devices.

A groundbreaking and lightweight method for detecting human falls, named CGNS-YOLO, has been developed. It aims to harmonize both detection precision and computational speed. This manuscript elucidates the following salient contributions:

The YOLOv5 neck network is enhanced by the integration of the GDCN module and the lightweight convolution technique, GSConv. This amalgamation results in model compression, refined feature extraction, and superior hardware compatibility.

The inclusion of the Normalization-based Attention Module (NAM) augments detection precision by judiciously highlighting critical data pertinent to fall detection.

The SIoU loss function is employed to expedite model convergence and further elevate detection accuracy.

## 2. Methods

### 2.1. Background of YOLOv5

The YOLOv5 model leverages deep learning for object recognition. It comprises five versions: YOLOv5n, YOLOv5s, YOLOv5m, YOLOv5l, and YOLOv5x. Distinct computational resource demands and detection accuracies characterize each version, largely attributable to variations in the width and depth of their residual structures. Nonetheless, all five maintain a consistent network architecture encompassing an input, a backbone, a neck, and a prediction head. In this context, we propose a novel method to render the model more lightweight, specifically targeting a reduction in floating-point operations, parameter count, and overall model size, while retaining its recognition efficacy.

For image input processing in YOLOv5s, three pivotal techniques are employed: mosaic data augmentation, adaptive image sizing, and anchor computation. The mosaic data augmentation enhances dataset diversity by merging four randomly sized images in a specific cropping and arrangement pattern, thus enriching background variability and increasing the presence of smaller objects—factors that elevate recognition precision. Adaptive image sizing entails the application of minimal black borders to original images, catering to their variable dimensions, after which they undergo proportional resizing to a uniform standard. The process of adaptive anchor computation involves gauging the disparity between predicted and actual bounding boxes, iteratively refining parameters to derive the optimal anchor boxes.

The backbone network of YOLOv5s incorporates three primary modules: Conv, C3, and Spatial Pyramid Pooling-Fast (SPPF). The Conv module, integrating convolutional layers, Batch Normalization (BN) layers [28], and the Silu activation function [29], functions as the foundational unit of YOLOv5s, systematically applying 2D convolution, normalization, and activation to the input data. The C3 module houses multiple bottleneck residual units, where the input to this residual structure traverses two convolutional layers before re-adding to its original value, thus conveying residual features without amplifying the output depth. The purpose of the SPPF module revolves around expanding the perceptual field, isolating vital contextual data, and addressing multi-scale challenges.

The neck network harnesses both the Path Aggregation Network (PANet) and C3 for optimal feature fusion. Initially, PANet utilizes upsampling to relay granular localization data from the foundational layers to their higher counterparts. Subsequently, a bottom-up Feature Pyramid Network (FPN) is deployed to transfer semantically reliable data from these higher echelons. This fusion by the PANet ensures that the data relayed from the neck to the head encapsulate both potent semantic details and refined localization cues, culminating in enhanced detection.

Lastly, the YOLOv5s framework adopts three detection layers, each producing feature vectors of distinct magnitudes. Each vector elucidates class probability, target score, and the spatial configuration of the target bounding box.

### 2.2. CGNS-YOLO Network Architecture Design

**Figure 1:**
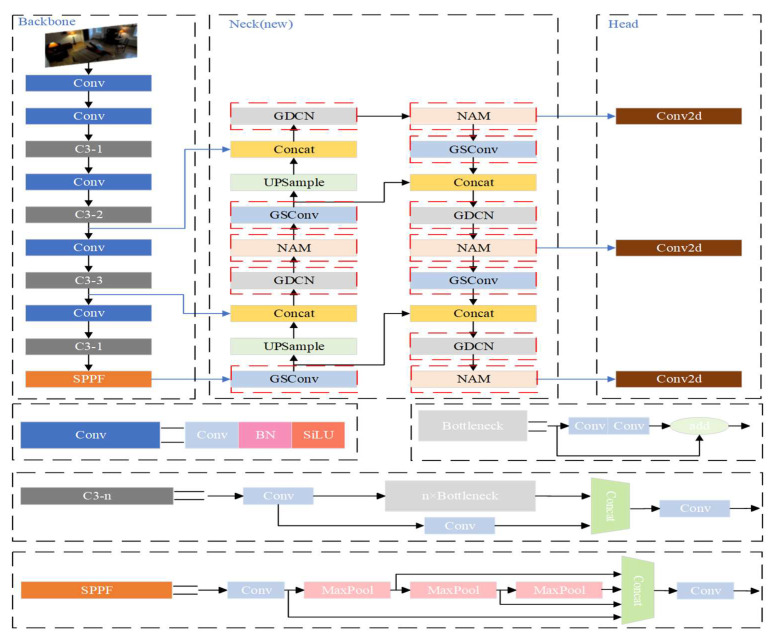
The architecture of CGNS-YOLO.

We introduce the CGNS-YOLO network, a lightweight architecture tailored to human fall detection, which builds upon the YOLOv5s framework. While retaining accuracy, the CGNS-YOLO network necessitates reduced computational resources and demonstrates a decrease in algorithmic complexity relative to its predecessor. A depiction of the CGNS-YOLO network architecture can be found in Figure 1, where the red dashed box indicates the part of the algorithm that has been improved.

Enhancements to the original YOLOv5s structure are manifested in the CGNS-YOLO network through three pivotal modifications. First, the C3 and Conv modules present in the original YOLOv5s neck network are supplanted by the more streamlined GDCN and GSConv modules. These latter modules are tailored to diminish model intricacy, bolster feature extraction efficacy, and maintain hardware adaptability. Second, the incorporation of the Normalization-based Attention Module (NAM) into the neck network accentuates limb-centric details, fortifying the network’s competence in deciphering human motion patterns and ensuring robust human action recognition across diverse backgrounds. Finally, a transition in the network’s training loss function from the Generalized Intersection over Union (GIou) to the SIoU loss function is instituted, facilitating swifter model convergence and augmented recognition precision throughout the training phase.

#### 2.2.1. Replacing C3 Modules in the Neck Network with the GDCN Modules

**Figure 2:**
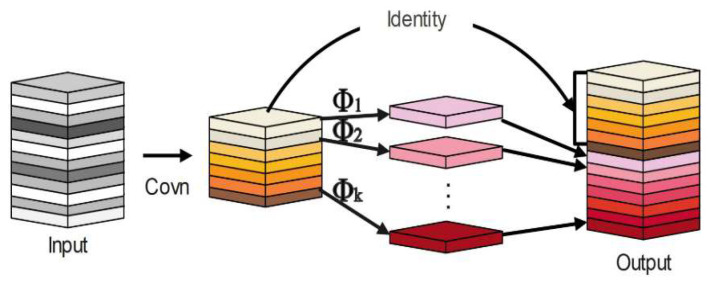
Ordinary Ghost module.

Han et al. [30] presented an innovative lightweight module, termed “Ghost” derived from the GhostNet architecture. Notably, this module can produce an augmented number of feature maps, utilizing fewer parameters and computations. For an input feature $X\in R^{H\times W\times C}$, where H, W,  C denote the width, height, and number of channels of the feature map, respectively, the operation of the Ghost module proceeds in the following sequence. A collection of feature maps is initially extracted via standard convolution, as detailed in Formula (1). These feature maps subsequently undergo a linear transformation to yield additional or “ghost” feature maps derived from the preceding maps. The primary and the “ghost” feature maps are then concatenated along a designated dimension, resulting in the final output, as articulated in Formula (2). The core motivation behind the Ghost module’s design is to curtail the computational overhead associated with traditional convolutions without compromising on accuracy. This operational framework is graphically depicted in Figure 2.

(1) $$Y\prime =X*F_{1\times 1}$$

(2) $$Y=Concat([Y\prime ,Y\prime *F_{dp}])$$

In Formula (1), ∗ denotes the convolution operation, $F_{1\times 1}$ is the point-wise convolution, and $Y\prime \in R^{H\times W\times C}$ represents the intrinsic features, whose sizes are usually smaller than the original output features. Then, cheap operations are used to generate more features based on the intrinsic features. The two parts of features are concatenated along the channel dimension. In Formula (2), $F_{dp}$ is the depth-wise convolutional filter, and $Y\in R^{H\times W\times C_{\mathrm{out}}}$ is the final output feature. Although the Ghost module has the potential to significantly reduce computational cost, its ability to represent features is limited by the fact that convolutional operations model only local information within a window. In the GhostNet architecture, low-cost operations such as 3 × 3 depth-wise convolution capture half of the features to preserve spatial information, and the remaining features are obtained by 1 × 1 point-wise convolutions without exchanging information with other pixels. However, this approach can lead to a weaker representation of spatial information, which may hinder further performance improvements.

To address the limitations of the Ghost module and improve the representation of spatial information, we introduce the Decoupled Fully Connected (DFC) [31] attention mechanism. Given an input feature $Z\in R^{H\times W\times C}$, it can be considered as HW tokens, i.e., $Z_{i}\in R^{C},Z=\{z_{11},z_{12},...,z_{HW}\}$. One way to implement an attention graph using an FC layer is shown in Formula (3):

(3) $$a_{hw}=\sum_{h\prime ,w\prime }F_{hw},h\prime ,w\prime ⊗Z_{h\prime ,w\prime }$$

In Formula (3), ⊗ is element-wise multiplication, $F^{HW\times H\times W}$ represents the learnable weights in the FC layer, and $A=\{a_{11},a_{12},...,a_{HW}\}$ is the generated attention map. Since all locations (represented in ∑h′,w′) contribute to the computation of the attention output $a_{hw}$ for each location, the global information is captured by combining all tokens using learnable weights. In addition, since CNN features are 2D, the FC layer can be computationally simplified by exploiting this 2D structure, as shown through the decomposition in Formula (3). Specifically, Formula (3) can be decomposed into two FC layers that aggregate features along the horizontal and vertical directions, respectively. This can be expressed as:

(4) $${a\prime }_{hw}=\sum_{h\prime =1}^{H}F_{h,h\prime ,w}^{H}⊗Z_{h\prime ,w\prime },h=1,2\ldots ,H,w=1,2,\ldots ,W$$

(5) $$a_{hw}=\sum_{w\prime =1}^{w}F_{w,hw\prime }^{W}⊗{a\prime }_{hw\prime },h=1,2\ldots ,H,w=1,2,\ldots ,W$$

**Figure 3:**
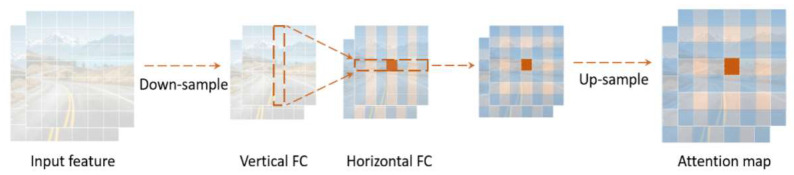
Information flow diagram of DFC.

The above formula uses $F^{H}$ and $F^{W}$ as transformation weights. To capture long-range correlations in two directions, the original feature *Z* is successively processed through Formulas (4) and (5) using the DFC attention mechanism, as shown in Figure 3. The process begins with the original feature *Z* as the input. Then, Formulas (4) and (5) are sequentially applied to the features for capturing long-range correlations in two directions. As evident from Figure 3, this is the flow of information that the DFC attention mechanism emphasizes. Formulas (4) and (5) are the general formulation of the DFC attention mechanism that aggregates pixels along the horizontal and vertical directions, respectively. Sharing a portion of the transformation weights makes it feasible to use convolutions to efficiently implement the DFC attention mechanism, avoiding the time-consuming tensor-reshaping and transposition operations that may affect inference speed. Decoupling the filter size from the feature map size can help to handle input images of varying resolutions. To be specific, two depthwise convolutions are applied sequentially to the input feature with kernel sizes 1×KH and KW×1. The decoupling of horizontal and vertical transformations reduces the computational complexity of the attention module to $O(H^{2}W+HW^{2})$.

**Figure 4:**
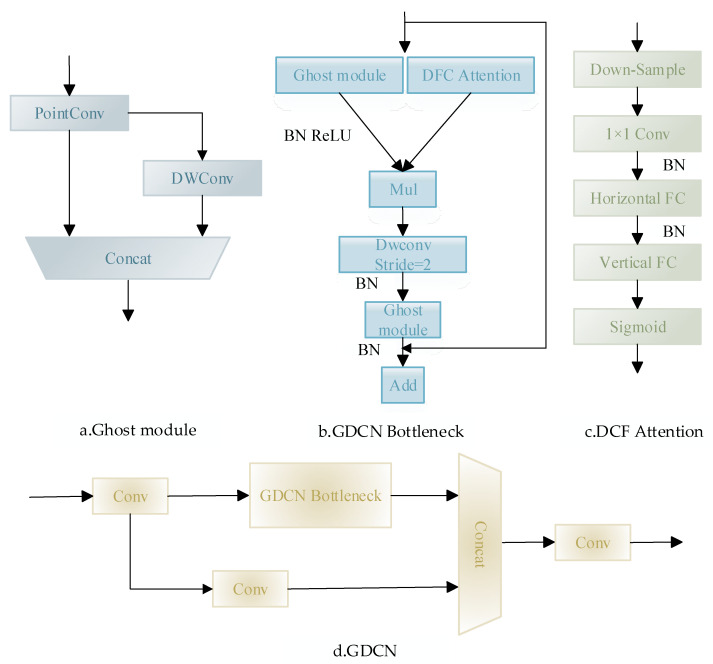
The architecture of the GDCN module.

We present the GDCN module, leveraging the Decoupled Fully Connected (DFC) attention mechanism. Drawing inspiration from both the Ghost module and the DFC attention mechanism, Figure 4 illustrates the design and intricacies of the GDCN module. Significantly, the GDCN Bottleneck, an innovative fusion of the Ghost module and the DFC attention mechanism, supersedes the conventional C3 Bottleneck within the neck network. This integration births the novel GDCN network. The inception of the GDCN network leads to a reduction in computational demands, a decrease in model dimensions, and a substantial improvement in detection precision. This pioneering strategy harnesses the combined potential of the Ghost module and the DFC attention mechanism, ultimately establishing the pivotal GDCN module, which amplifies the overall effectiveness of the GDCN network.

#### 2.2.2. Substituting Conv Modules in the Neck Network with the GSConv Modules

Although the Standard Convolution (SC) [32] has demonstrated enhanced accuracy within the network, its associated computational cost is considerable, potentially compromising the real-time demands of fall detection tasks. This has necessitated the contemplation of a lightweight convolutional design. Currently, the Depth-wise Separable Convolution (DSC) [33] is predominantly adopted in lightweight architectures due to its efficacy in parameter and floating-point operation reduction. However, a limitation of the DSC is its channel information separation during computations. To address this, we introduce GSConv [34], a novel convolutional approach. GSConv amalgamates the features of the SC, the DSC, and shuffle, aiming to bring the output accuracy of the DSC in closer alignment with that of the SC.

The GSConv module primarily consists of the SC module, DSC module, Concat module, and shuffle module. Formula (6) provides the mathematical expression for it. In this formula, $f_{shuffle}$ denotes the channel mixing and washing operation, $f_{sc}$ denotes the standard convolution, and $f_{dsc}$ denotes the deep separable convolution.

(6) $$X_{out}=f_{shuffle}(cat(f_{conv}(X_{in}),f_{dsc}(f_{conv}(x_{in}))))$$

**Figure 5:**
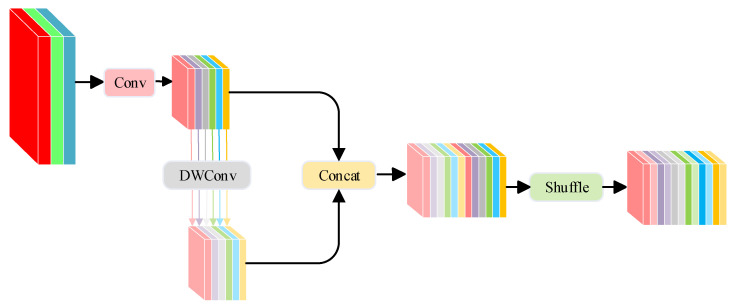
The architecture of the GSConv module.

As illustrated in Figure 5, the GSConv module adopts a shuffle strategy to disseminate the information derived from the SC throughout the data generated by the DSC. This shuffle technique is characterized by a uniform mixing approach, ensuring that information from the SC is seamlessly integrated into the DSC’s output through the consistent swapping of local feature details across different channels. The procedure is streamlined, with no added computational demands.

In the context of fall detection tasks, convolutional neural networks frequently apply image transformations to facilitate predictive modeling. These transformations systematically reorganize spatial data into channel-based representations. However, each transformation inherently leads to a trade-off: as spatial dimensions decrease, channel dimensions expand, potentially resulting in a loss of vital semantic information. Traditional standard convolution computation (SC) retains essential inter-channel correlations, while the depthwise separable convolution (DSC) severs these connections. The GSConv module is meticulously designed to combine the advantages of both the SC and DSC, aiming to preserve these interconnections while optimizing computational efficiency. Consistently implementing GSConv across multiple network layers results in increased network depth, enhanced resilience to data flow, and longer inference times. As feature maps progress through the network and reach the “neck” phase, they undergo elongation, characterized by maximized channel dimensions and minimized spatial dimensions. Consequently, transformations become relatively muted. Thus, it is prudent to employ GSConv exclusively within the neck network. In this phase, GSConv finds its optimal position for processing amalgamated feature maps, thanks to the reduced redundancy and the minimal need for further compression. This strategic use of GSConv within the neck network ensures that the network maintains its efficiency and effectiveness while addressing the unique requirements of fall detection tasks.

#### 2.2.3. Embedding the Normalized Spatial Attention Module (NAM) into the Neck Network

Lightweight networks offer the advantage of enabling efficient real-time object detection in fall detection tasks, albeit occasionally at the cost of a diminished expressive capability. Incorporating attention mechanisms into such networks can amplify their expressive prowess by emphasizing visual or motion features pertinent to falls, thereby heightening accuracy. These attention mechanisms not only bolster accuracy but also reduce computational overhead and enhance network efficiency by filtering out less crucial features. Thus, marrying lightweight networks with attention mechanisms provides a synergistic boost to both the accuracy and real-time operability of fall detection models.

In recent times, attention mechanisms have garnered significant research attention due to their ability to assist deep neural networks in filtering out less crucial pixels or channels. Historically, the focus of research was primarily on deploying attention operations to seize salient features by adeptly leveraging the mutual information dispersed across various feature dimensions. However, the pivotal role of weight-contribution factors that could further attenuate non-essential channels or pixels remained underexplored. Addressing this gap, Liu et al. [35] integrated these weight-contribution factors to enhance attention mechanisms, leading to the formulation of an adept attention mechanism: the Normalization-based Attention Module (NAM).

**Figure 6:**
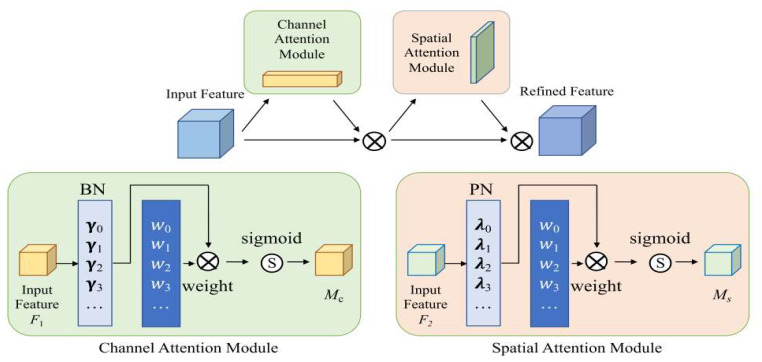
The architecture of the NAM module.

Built on the foundational principles of the Convolutional Block Attention Module (CBAM) [36] framework, the NAM attention mechanism stands out as an efficient and streamlined module. As depicted in Figure 6, it boasts a refined configuration of both channel and spatial attention sub-modules. Central to NAM’s design is its use of weights derived from image channel and spatial attributes to gauge the significance of image features, thus mitigating irrelevant channels and pixel data. In a departure from traditional attention designs, the NAM module employs the scaling coefficients of Batch Normalization (BN) to signify weight importance, as articulated in Formula (7). This approach obviates the need for intricate procedures involving dense and convolutional layers, preserving the module’s lightweight architecture and simultaneously bolstering network detection efficiency.

(7) $$B_{out}=BN(B_{in})=\gamma \frac{B_{in}-\mu b}{\sqrt{\sigma_{b}^{2}}}+\beta$$

Formula (7) shows that μb and σb represent the mean and standard deviation of each batch b, respectively, and γ and β represent the trainable scale and displacement parameters, respectively.

The channel attention module uses the γ-normalized correlation weights $W_{\lambda }$ method to give more attention to important channels while suppressing less informative weights. Let $F_{1}\in R^{H\times W\times C}$ be the input feature map, where H, W, C represent the height, width, and number of channels, respectively. The output of the channel attention model $M_{c}$ can be expressed as Formula (8):

(8) $$M_{c}=sigmoid(W_{\gamma }(BN(F_{1})))$$

The design approach used in the channel attention module has also been applied in the spatial attention module. Here, the pixels in the spatial dimensions go through *BN* processing, which is also known as Pixel Normalization (PN). PN focuses on the more informative pixels and adjusts the associated weights $W_{\lambda }$ accordingly based on the scaling factor λ. If $F_{2}\in R^{H\times W\times C}$ is the input feature map, where H, W, C denote the height, width, and number of channels, respectively; then, the output of the spatial attention model, $M_{s}$, can be expressed as Formula (9):

(9) $$Ms=sigmoid(W\lambda (BN(F_{2})))$$

The Normalization-based Attention Module (NAM) introduces a regularization to the loss function to suppress less significant weights, as expressed in Formula (10):

(10) $$L\mathrm{oss}=\sum_{\left(x,y\right)}l(f(x,W),y)+p\sum g(\gamma )+p\sum g(\lambda )$$

In Formula (10), l(⋅) and g(⋅) denote the loss function and l1 parametric penalty function, respectively. x and y represent the input and output, respectively, W is the network weight, and p is the equilibrium penalty factor.

In object detection networks, the “neck” component plays a pivotal role as a crucial bridge, connecting the backbone to the head of the architecture. In the context of fall detection, this neck component serves the essential function of extracting pertinent features from the backbone and subsequently refining them for precise target prediction in the head. In scenarios where intricate backgrounds pose challenges, incorporating appropriate attention mechanism modules within the neck can significantly enhance the model’s focus on the intended target, consequently improving the accuracy of fall detection. Therefore, in our study, we strategically integrated NAM modules into the 14th, 19th, 23rd, and 27th layers of the CGNS-YOLO network. This strategic placement was designed to enhance both channel and spatial data within the feature fusion layers. This approach intensifies the model’s responsiveness to visual and motion characteristics that are critical for fall detection, especially in complex environments. This enhancement leads to an improved discriminative capacity and robustness of the model, making it more effective in challenging settings. The incorporation of NAM modules at these specific network layers empowers the model to better capture and distinguish critical features for fall detection, ultimately contributing to its overall performance and reliability.

#### 2.2.4. The Role of SCYLLA Intersection over Union (SIoU) Loss in Improving Model Efficiency

In object detection, standard loss functions predominantly hinge on bounding box regression, utilizing a suite of metrics to discern discrepancies between predicted and ground truth boxes. This suite encapsulates metrics such as distance, overlap area, and aspect ratios. Prominent loss functions in the domain include Intersection over Union (IoU), Generalized Intersection over Union (GIoU) [37], Distance Intersection over Union (DIoU) [38], and Complete Intersection over Union (CIoU) [38]. Of these, the IoU loss function, quantifying the ratio of the intersection to the union of predicted and ground truth boxes, remains a staple in object detection. Although the IoU loss effectively gauges the overlapping region between two boxes, it remains silent on issues like the inter-box distance or the precision of overlap magnitude. The GIoU loss function, in its evolution, introduces the paradigm of outer and intersection bounding boxes, adeptly addressing both overlap accuracy and the non-overlapping zones. Notably, the GIoU loss reverts to the IoU loss under circumstances where the ground truth box envelops the predicted counterpart. To surmount GIoU’s constraints, the DIoU loss augments it by optimizing the normalized distance between the centroids of two boxes, thus expediting convergence. The CIoU loss function, building on the DIoU’s foundation, boosts precision via the incorporation of an aspect ratio penalty term.

While the aforementioned loss functions effectively capture differences between predicted and ground truth bounding boxes, they often overlook variations in orientation between these entities. This oversight can lead to prolonged convergence, reduced efficiency, or even model stagnation. To address this limitation, our research introduces the SCYLLA Intersection over Union (SIoU) [39] loss function. In addition to conventional metrics like distance and overlap area, the SIoU accounts for the orientation disparity between bounding boxes by introducing an innovative angle penalty term. This angle penalty plays a crucial role during model training, guiding predicted boxes toward the closest coordinate axis, thus improving their alignment with the ground truth. Furthermore, the angle penalty mandates the regression of a single coordinate, either in the X or Y dimensions, effectively reducing the number of trainable parameters. The incorporation of the SIoU loss results in a substantial enhancement in the precision and efficiency of the CGNS-YOLO fall detection model. The SIoU loss is composed of four key components: the angle cost, distance cost, shape cost, and IoU cost, each contributing to its ability to address orientation disparities and improve the model’s performance. This use of the SIoU loss function provides a more comprehensive and effective way to assess bounding box alignment, leading to improved precision and efficiency in the context of fall detection within the CGNS-YOLO model.

**Figure 7:**
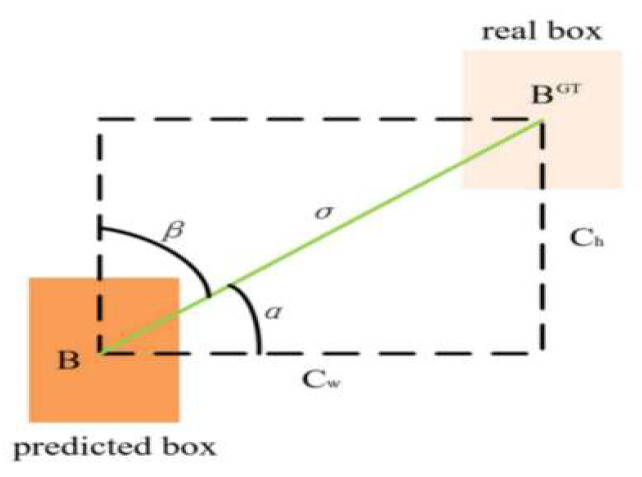
The scheme for calculating the contribution of the angle cost to the loss function.

The angle cost formula Λ is defined as Formula (11). As shown in Figure 7, the angle cost is 0 when the angle is $\alpha =\frac{\pi }{2}$ or α=0. During the training process, α is minimized when $\alpha <\frac{\pi }{4}$, and β is minimized otherwise.

(11) $$\Lambda =\cos[2\times \sin^{2}(\arcsin\frac{c_{h}}{\sigma }-\frac{\pi }{4})]$$

(12) $$\sigma =\sqrt{(b_{cx}^{gt}-b_{cx}{)}^{2}+(b_{cy}^{gt}-bcy{)}^{2}}$$

(13) $$c_{h}=\max(b_{cy}^{gt},b_{cy})-\min(b_{cy}^{gt},b_{cy})$$

In the formula, $c_{h}$ is the difference in height between the centroids of the real box and the predicted box, σ is the distance between the centroids of the real box and the predicted box, $\left(b_{cx}^{gt},b_{cy}^{gt}\right)$ is the center coordinate of the real box, and $\left(b_{cx},b_{cy}\right)$ is the center coordinate of the predicted box.

The distance cost formula Δ is defined as Formula (14).

(14) $$\Delta =2-e^{-(2-\Lambda )(\frac{b_{cx}^{gt}-b_{cx}}{c_{wl}}{)}^{2}}-e^{-(2-\Lambda )(\frac{b_{cy}^{gt}-b_{cy}}{c_{hl}}{)}^{2}}$$

In Formula (14), $\left(c_{wl},c_{hl}\right)$ represents the width and height of the minimum outer rectangle that includes both the real and predicted bounding boxes.

The shape cost formula Ω is defined as Formula (15).

(15) $$\Omega =(1-e^{-\frac{\left|w-w^{gt}\right|}{\max(w,w^{gt})}}{)}^{\theta }+(1-e^{-\frac{\left|h-h^{gt}\right|}{\max(h,h^{gt})}}{)}^{\theta }$$

In Formula (15), (w,h) represent the width and height of the predicted frame, $\left(w^{gt},h^{gt}\right)$ represent the width and height of the real frame, and θ indicates the level of importance given to the shape loss.

In summary, the SIoU loss function can be defined as Formula (16).

(16) $$Loss_{SIoU}=1-IoU+\frac{\Delta +\Omega }{2}$$

## 3. Experiments

### 3.1. Data Set

**Figure 8:**
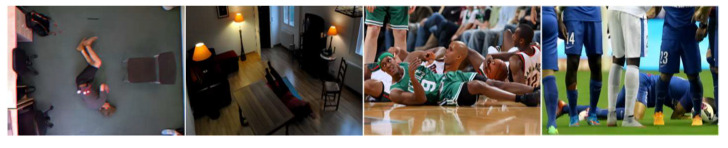
Typical fall detection images.

**Figure 9:**
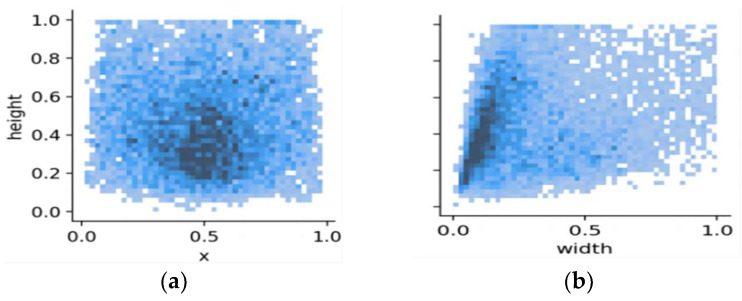
Image division: Where (**a**) is the target center-of-mass location distribution, and (**b**) is the image size distribution.

In addressing the constraints posed by the limited diversity and sample size of extant publicly accessible fall detection datasets, this study employed a comprehensive dataset amalgamated from various sources. Specifically, this amalgamated dataset integrates several publicly available datasets, encompassing the UR Fall Detection Dataset, Le2i Fall Detection Dataset, Multicam, and the AI Studio platform’s Fall Detection Dataset. To bolster the model’s generalizability, the dataset amalgamation considers multifaceted dimensions, such as varied camera perspectives, lighting nuances, obstructions from objects and pedestrians, multi-individual falls, and varied fall orientations and postures, as well as a diverse demographic comprising different age brackets and physiques. Moreover, to enhance dataset robustness and utility, a considerable collection of ambiguous fall instances was gathered to serve as negative samples. Data refinement and augmentation methods were leveraged to equilibrate the dataset labels. The culminated dataset encompasses ten thousand images partitioned into training, validation, and test subsets at an 8:1:1 distribution. Figure 8 depicts sample visuals from the dataset, representing quintessential fall detection contexts, whereas Figure 9 delineates the dispersion of object centroids and image dimensions within the fall detection visuals.

### 3.2. Experimental Process

A deep learning framework, developed on PyTorch 1.13.0, was employed for the training and evaluation of the fall detection model. Experiments were conducted on a system equipped with an AMD Ryzen9 5900HX CPU and an NVIDIA GeForce RTX 3060 GPU, utilizing Windows 10 operating system. The development environment comprised PyCharm 2022.1.2 and Python 3.8. The model is designed to process input images with a resolution of 640 × 640 pixels. For training parameters, a batch size of 16 was selected, training iterations (epochs) were capped at 200, momentum was fixed at 0.937, the initial learning rate was set to 0.001, and a decay coefficient of 0.9 was implemented.

### 3.3. Evaluation Criteria

In the present investigation, the efficacy of the proposed GDCN fall detection algorithm is quantified using precision (P). Recall (R) gauges the detection of positive samples across all datasets. The detector’s competency in individual categories is ascertained via the average precision (AP), and the mean average precision (mAP) represents the arithmetic mean across all AP categories. Equations (17)–(20) delineate the definitions of P, R, AP, and mAP, respectively. The complexity associated with the algorithm or model is evaluated based on parameters (Params) and floating-point operations (FLOPs).

(17) $$P=\frac{TP}{TP+FP}\times 100\%$$

(18) $$R=\frac{TP}{TP+FN}\times 100\%$$

(19) $$AP=\int_{0}^{1}P(R)dR\times 100\%$$

(20) $$mAP=\frac{\sum_{i=1}^{k}AP}{k}$$

Specifically, TP represents the number of positive samples correctly predicted, TN represents the number of negative samples correctly predicted, FP represents the number of negative samples classified as positive, FN represents the number of positive samples classified as negative, and k represents the number of categories.

### 3.4. Experimental Results and Analysis

To evaluate the efficacy of the CGNS YOLO algorithm, we designed two distinct sets of comparative experiments. The initial set juxtaposed the original YOLOv5s model with its enhanced counterparts, focusing on performance and accuracy metrics. In contrast, the subsequent set compared the refined models with both the comprehensive YOLO series and prevailing object detection algorithms, specifically assessing performance and accuracy parameters.

**Figure 10:**
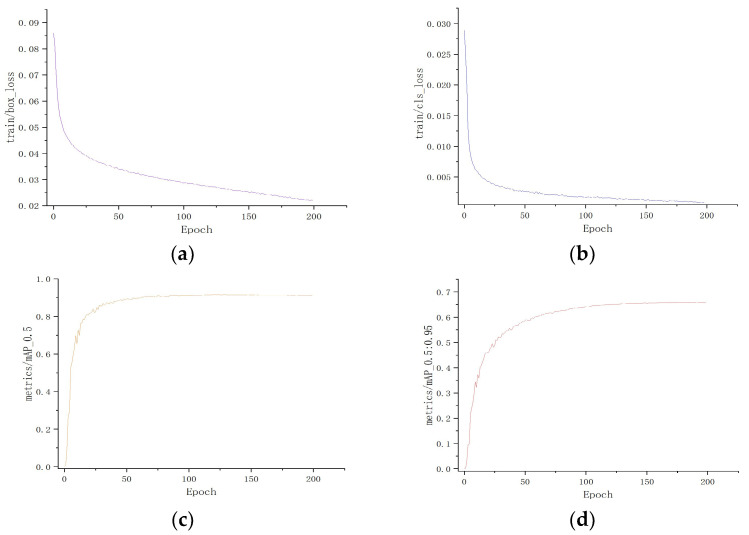
Model training result curves: (**a**) is the train/box_loss curve, (**b**) is the train/cls_loss curve, (**c**) is the mAP0.5 curve, and (**d**) is the mAP0.5:0.95 curve.

Figure 10 delineates the training outcomes. Analyzing both loss curves reveals a swift decline in loss values during the preliminary training phases. However, with an escalation in the training epochs, this decline becomes more gradual, culminating in stabilization around a specific threshold. At approximately the 20th epoch, a stability in losses is noted, signifying effective model convergence and the absence of overfitting throughout the training regimen.

Similarly, observing the dual accuracy curves, there is an evident swift surge in accuracy during the initial training phases. However, with the progression of training epochs, this ascent becomes moderate, causing the accuracy to oscillate around a set threshold. By approximately the 50th epoch, accuracy reaches a plateau, indicating that the models have attained an optimal accuracy benchmark.

#### 3.4.1. Improved Content Comparison Experiment

A series of ablation experiments were executed to assess the effectiveness of various modifications on the original network. The outcomes of these experiments are tabulated in Table 1. In this context, Improved Model 1 employs GDCN in the neck network as a substitute for C3; Improved Model 2 introduces GSConv to the neck network, replacing the traditional Conv; Improved Model 3 is characterized by the inclusion of NAM within the neck network; Improved Model 4 pertains to adjustments made to the loss function; and Improved Model 5 amalgamates all the enhancements mentioned above into the foundational network.

As delineated in Table 1, by introducing the GDCN module into the original YOLOv5s network configuration, there is a notable reduction in Params by 23.4% and FLOPs by 33.3%. Furthermore, there is a marginal elevation in the model’s mAP by 0.5%. The primary attributing factor is the replacement of the C3 module with GDCN, which facilitates the production of augmented feature maps via linear operations. Such enriched feature information often fosters a holistic comprehension of the input attributes. Consequently, this research integrates the streamlined structure of GDCN into the YOLOv5s network without compromising detection precision. By substituting the Conv configuration with GSConv in the YOLOv5s neck network, the model’s mAP remains stable, but there is a 6% decrease in Params. Incorporating NAM elevates the mAP by a modest 0.4% relative to the baseline model, with negligible changes in both Params and FLOPs. After refining the loss function, there is a 0.3% enhancement in mAP. Upon integrating all four aforementioned modifications, the model, when juxtaposed with the foundational YOLOv5s network, displays an mAP increment of 1.2%, a 20.3% reduction in Params, and a 29.6% decrement in FLOPs.

The analysis indicates that the new neck network does effectively strengthen the feature extraction capabilities of the backbone network, facilitating a precise capturing of intricate details. There is increased accuracy in identifying positive samples, thereby reducing false alarms. Furthermore, genuine positive samples can now be detected more thoroughly, thereby improving the overall detection performance of the model. This optimization has shown a positive impact across different IoU thresholds, significantly improving the model’s performance in various scenarios.

These findings substantiate that the enhanced YOLOv5s not only delivers a superior detection efficacy in fall detection contexts but also benefits from the incorporation of lightweight modules, leading to a reduced model intricacy.

#### 3.4.2. Comparison Experiment

To assess the efficacy of various lightweight models in fall detection, we selected nine representative lightweight network models for comparison: YOLOv3-Tiny, YOLOv4-Tiny, YOLOv5s, YOLOX-s, YOLOv7, YOLOv7-Tiny, YOLOv8s, DAMO-YOLO-T, and Faster R-CNN. These were juxtaposed with CGNS-YOLO for a thorough evaluation. Uniformity in evaluation was maintained as all models were trained and tested using the same dataset. Performance metrics encompassed mAP, Precision (P), Recall (R), Model Size, Params, and FLOPs. The outcomes of this fall detection comparison are encapsulated in Table 2.

The analysis of the results reveals that the CGNS-YOLO model, when compared with the aforementioned models, outperforms in terms of mAP, P, and R with scores of 91.3%, 90.4%, and 89.1%, respectively. Although the size, parameters, and FLOPs of CGNS-YOLO are greater than those of YOLOv4-Tiny, they are significantly smaller than those of the other models, registering at 11.3 MB, 5.1 M, and 11.2 G, respectively. In a more detailed comparison, CGNS-YOLO exhibited enhancements in mAP by 1.4%, 4.4%, 1.2%, 4%, 1.8%, 2.6%, 0.7%, 0.4%, and 10.2%; in P by 3.8%, 4.3%, 1.9%, 6.5%, 2.7%, 6.8%, 1.4%, 0.8%, and 9.9%; and in R by 3.9%, 7.1%, 1.5%, 8.7%, 6%, 3.2%, 0.8%, 0.3%, and 9.6%, respectively. Furthermore, relative to lightweight models like YOLOv3-Tiny, YOLOv5s, YOLOX-s, YOLOv7-Tiny, and YOLOv8s, CGNS-YOLO demonstrated reductions in model size by 35.4%, 20.98%, 66.9%, 8.1%, and 47.4%; in Params by 38.6%, 20.3%, 43.3%, 10.5%, and 54.5%; and in FLOPs by 13.8%, 29.6%, 58.1%, 15.2%, and 60.8%, respectively. Cumulatively, these findings underscore that the enhanced CGNS-YOLO model not only ensures outstanding detection accuracy but also significantly optimizes the lightweight attributes of the network.

### 3.5. Practical Scenario Testing

**Figure 11:**
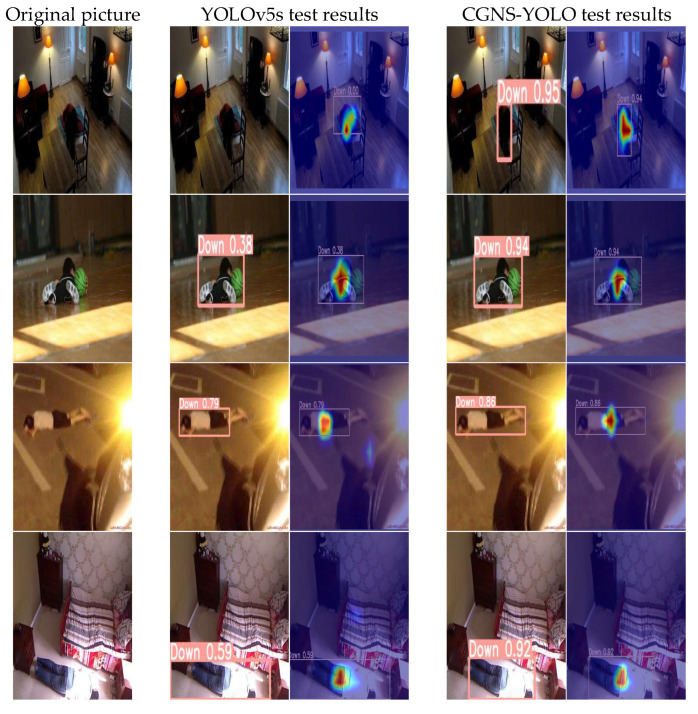
Different light conditions.

**Figure 12:**
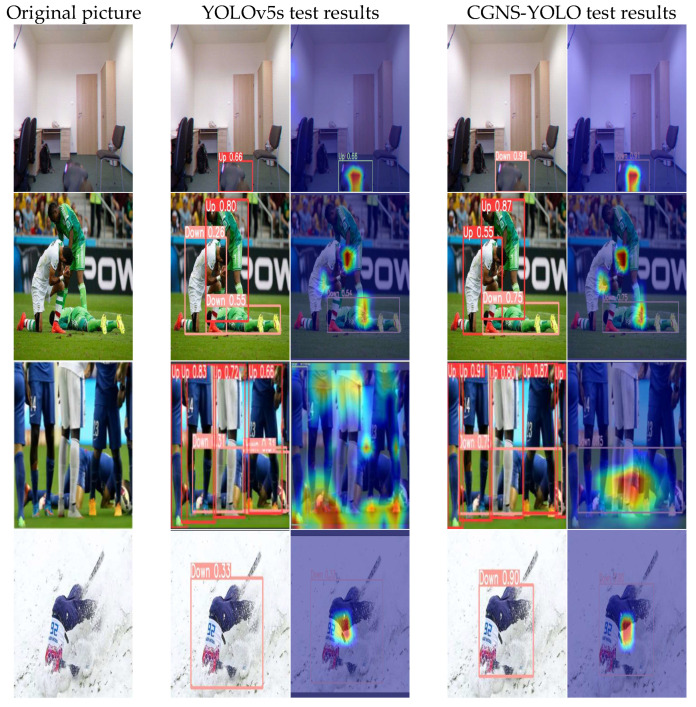
Occlusion scenarios.

This study undertook visual analyses to delineate the disparities in fall detection performance between the original YOLOv5s and the CGNS-YOLO algorithm across diverse settings, encompassing variable lighting conditions and occlusion instances. The culminating visual findings are portrayed in Figure 11 and Figure 12. Every test image set comprises three components: the left-most image presents the authentic photo, whereas the central pair of images depict the detection outcomes of the original YOLOv5s algorithm alongside their respective GradCam visualizations. In parallel, the rightmost pair illustrates the detection results of the CGNS-YOLO algorithm, complemented by their corresponding GradCam visualizations.

Figure 11 elucidates that the predominant challenge encountered by the original YOLOv5s in dimly lit cluttered environments is its inability to discern the target with precision. In juxtaposition, CGNS-YOLO demonstrates adeptness in pinpointing targets even in suboptimal lighting, with its predictive accuracy observing a marked enhancement, surging by over 50%. When subjected to intense luminous disruptions, the original YOLOv5s grapples with imprecise bounding box localization. However, the integration of CGNS-YOLO culminates in refined bounding box localization, registering a 20% surge in predictive accuracy. These enhancements compellingly underscore the preeminence of CGNS-YOLO in executing fall detection tasks across varied luminance contexts.

From the data presented in Figure 12, it becomes evident that the original YOLOv5s model encounters challenges in discerning true positives during occlusion scenarios. When the camera’s field of view experiences obstructions, the model is prone to misidentifying a falling individual as standing upright. Moreover, in situations where certain body parts are obscured, the model might mistakenly interpret a standing individual as falling or erroneously detect a single falling individual as two distinct entities. Such occlusions often misguide the model, leading to inaccurate target localization and a consequent decline in prediction precision. As a result, the bounding box localization becomes erratic, the network’s focus is dispersed, and pinpointing the primary target becomes problematic. In contrast, the CGNS-YOLO algorithm adeptly detects occluded subjects, markedly enhancing the prediction accuracy for fall states. The precision of bounding box localization witnesses notable improvement, with the network’s attention zeroing in on the primary subject. This effectively curtails instances of both false positives and false negatives. Evidently, the CGNS-YOLO model exhibits a pronounced advantage when addressing fall detection challenges in occluded environments.

After examining the experimental results from the before-and-after comparison, it is clear that the introduction of the new neck network significantly improved the model’s performance. The inclusion of GDCN substantially increased the network’s ability to adjust its sensory field, allowing for more effective target detail capture. Additionally, GSConv efficiently compressed the model’s size and reduced its overall complexity. Furthermore, the incorporation of NAM into the C3 system strengthened the model’s emphasis on crucial characteristics, resulting in improved accuracy for projecting frame placement and increased effectiveness in identifying obstructions.

From comprehensive experimental evaluations across diverse scenarios, it is unequivocally established that the CGNS-YOLO algorithm exhibits pronounced efficacy in numerous contexts. The algorithm not only enhances the accuracy of human fall detection but also notably augments the precision of bounding box localization. Moreover, it effectively focuses network attention, amplifies occlusion detection capabilities, and markedly diminishes the instances of false positives and negatives. Consequently, the CGNS-YOLO algorithm showcases superior performance, underscoring its profound advantages in myriad human fall detection applications.

## 4. Conclusions

We introduce CGNS-YOLO, a novel lightweight method for human fall detection, devised to address the computational challenges inherent in employing the conventional YOLOv5 for fall detection scenarios. The salient contributions of this work are enumerated as follows:

(1) The neck network of YOLOv5 was re-engineered employing GDCN and GSConv, which substantially curtailed the model’s parameters while enhancing both detection accuracy and speed.
(2) The integration of the NAM module augmented detection accuracy with negligible computational overhead.
(3) The adoption of the SIoU loss function expedited model convergence, concurrently elevating detection accuracy.
(4) CGNS-YOLO displayed superior performance when juxtaposed with prevalent state-of-the-art lightweight algorithms, including YOLOv3-tiny, YOLOv4-tiny, YOLOv5s, YOLOv7-tiny, and YOLOv8s.

Empirical results derived from the test set revealed that CGNS-YOLO achieved an mAP of 91.3%, P of 90.4%, and R of 89.1%, with model dimensions, parameters, and FLOPs of 11.4 MB, 5.1 M, and 11.2 G, respectively. When contrasted with the original YOLOv5s network, CGNS-YOLO exhibited reductions in model size, parameters, and FLOPs of 21.0%, 20.3%, and 29.6%, respectively, and simultaneously showed a 1.2% mAP increment. The empirical outcomes and subsequent analysis underscore that CGNS-YOLO possesses a more streamlined architecture, diminished complexity, and superior detection accuracy, aptly satisfying real-time detection prerequisites. In comparison with extant models, CGNS-YOLO manifests a superior detection efficacy with a lesser computational footprint, thereby economizing memory and computational demands on platforms and enhancing hardware compatibility. In summary, CGNS-YOLO bolsters the precision and efficiency of elderly fall detection, embodying tangible merit in real-time fall detection and alert mechanisms. Prospective investigations might pivot toward deploying CGNS-YOLO in resource-limited embedded fall detection systems and further honing the presented algorithm for pragmatic utilization.

## Figures and Tables

**Table 1:** Ablation experiments with different improvement strategies.

Models	GDCN	GSConv	NAM	SIoU	Map (0.5)	Params (M)	FLOPs (G)
YOLOv5s	×	×	×	×	90.1	6.4	15.9
YOLOv5s_1	√	×	×	×	90.6	4.9	10.6
YOLOv5s_1	×	√	×	×	90.1	6.0	15.7
YOLOv5s_1	×	×	√	×	90.5	6.6	16.3
YOLOv5s_1	×	×	×	√	90.4	6.4	15.9
GDCN-YOLO	√	√	√	√	91.3	5.1	11.2

**Table 2:** Comparison experiments of fall detection results with different lightweight models.

Models	Map (0.5)	P	R	Model Size (MB)	Params (M)	FLOPs (G)
YOLOv3-Tiny	88.9	86.6	85.2	17.5	8.3	13.0
YOLOv4-Tiny	86.9	86.1	82.0	6.3	2.9	6.4
YOLOv5s	90.1	88.5	87.6	14.3	6.4	15.9
YOLOX-s	87.3	83.9	80.4	34.1	9.0	26.7
YOLOv7	89.5	87.7	83.1	75	35.3	105.1
YOLOv7-Tiny	88.7	83.6	85.9	12.3	5.7	13.2
YOLOv8s	90.6	89.0	88.3	21.5	11.2	28.6
DAMO-YOLO-T	90.9	88.6	88.8	34.9	8.5	18.1
Faster R-CNN	81.1	80.5	79.5	132.1	66.3	152.1
CGNS-YOLO	91.3	90.4	89.1	11.3	5.1	11.2

## Data Availability

The Fall detection Dataset was obtained from http://falldataset.com, accessed on 3 October 2022. The UR Fall Detection Dataset was obtained from http://fenix.ur.edu.pl/~mkepski/ds/uf.html, accessed on 3 October 2022. The Multiple-cameras fall dataset was obtained from http://www.iro.umontreal.ca/~labimage/Dataset/, accessed on 9 October 2022.

## References

[1] Delgado-Escano R., Castro F.M., Cozar J.R., Marin-Jimenez M.J., Guil N., Casilari E. (2020). A cross-dataset deep learning-based classifier for people fall detection and identification. Comput. Methods Programs Biomed..

[2] Girshick R. (2015). Fast R-CNN. Proceedings of the 2015 IEEE International Conference on Computer Vision (ICCV).

[3] Dai J., Li Y., He K., Sun J. (2016). R-fcn: Object detection via region-based fully convolutional networks. Adv. Neural Inf. Process. Syst..

[4] He K., Gkioxari G., Dollár P., Girshick R. (2017). Mask R-CNN. Proceedings of the 2017 IEEE International Conference on Computer Vision (ICCV).

[5] Cai Z., Vasconcelos N. (2018). Cascade R-CNN: Delving into high quality object detection. Proceedings of the 2018 IEEE/CVF Conference on Computer Vision And Pattern Recognition.

[6] Redmon J., Divvala S., Girshick R., Farhadi A. (2016). You only look once: Unified, real-time object detection. Proceedings of the 2016 IEEE Conference on Computer Vision and Pattern Recognition (CVPR).

[7] Redmon J., Farhadi A. (2017). YOLO9000: Better, faster, stronger. Proceedings of the 2017 IEEE Conference On Computer Vision And Pattern Recognition.

[8] Redmon J., Farhadi A. (2018). Yolov3: An incremental improvement. arXiv.

[9] Bochkovskiy A., Wang C.Y., Liao HY M. (2020). Yolov4: Optimal speed and accuracy of object detection. arXiv.

[10] Liu W., Anguelov D., Erhan D., Szegedy C., Reed S., Fu C.Y., Berg A.C. (2016). Ssd: Single shot multibox detector. Proceedings of the 14th European Conference on Computer Vision.

[11] Feng Y.P., Guan Y.Y., Yang X.R., Liu N., Wang Z.H. (2021). Real-time pedestrian detection algorithm fused with attention mechanism. Electron. Meas. Technol..

[12] He Z.F., Chen G.C., Chen J.S., Zhang Y.H. (2022). Multi-Scale Feature Fusion Lightweight Real-Time Infrared Pedestrian Detection at Night. Chin. J. Lasers.

[13] Hu J., Shen L., Sun G. (2018). Squeeze-and-excitation networks. Proceedings of the 2018 IEEE/CVF Conference on Computer Vision and Pattern Recognition Workshops (CVPRW).

[14] Lin T.Y., Dollár P., Girshick R., He K., Hariharan B., Belongie S. (2017). Feature pyramid networks for object detection. Proceedings of the 2017 IEEE Conference on Computer Vision and Pattern Recognition (CVPR).

[15] Liu S., Qi L., Qin H., Shi J., Jia J. (2018). Path aggregation network for instance segmentation. Proceedings of the 2018 IEEE/CVF Conference on Computer Vision and Pattern Recognition.

[16] Zhu X., Lyu S., Wang X., Zhao Q. (2021). TPH-YOLOv5: Improved YOLOv5 based on transformer prediction head for object detection on drone-captured scenarios. Proceedings of the 2021 IEEE/CVF International Conference on Computer Vision Workshops (ICCVW).

[17] Wang Z., Jin L., Wang S., Xu H. (2022). Apple stem/calyx real-time recognition using YOLO-v5 algorithm for fruit automatic loading system. Postharvest Biol. Technol..

[18] Zhao J., Zhang X., Yan J., Qiu X., Yao X., Tian Y., Zhu Y., Cao W. (2021). A wheat spike detection method in UAV images based on improved YOLOv5. Remote Sens..

[19] Chen T., Ding Z., Li B. (2022). Elderly Fall Detection Based on Improved YOLOv5s Network. IEEE Access.

[20] Peng J., He Y., Jin S., Dai H., Peng F., Zhang Y. (2022). Improved YOLOv5 Method for Fall Detection. Proceedings of the 2022 IEEE 17th Conference on Industrial Electronics and Applications (ICIEA).

[21] Chen S.Q., Wang C.Q., Zhou Y.J. (2022). A Pedestrian Detection Method Based onYOLOv5s and Image Fusion. Electron. Opt. Control..

[22] Chen Y., Alifu K., Lin W.L. (2022). CA-YOLOv5 for Crowded Pedestrian Detection. Comput. Eng. Appl..

[23] Fu N., Liu D., Cheng X., Jing Y., Zhang X. (2021). Fall detection algorithm based on lightweight OpenPose model. Transducer Microsyst. Technol..

[24] Nguyen H.C., Nguyen T.H., Nowak R., Byrski J., Siwocha A., Le V.H. (2022). Combined YOLOv5 and HRNet for high accuracy 2D keypoint and human pose estimation. J. Artif. Intell. Soft Comput. Res..

[25] Li W., Zhang L., Wu C., Cui Z., Niu C. (2022). A new lightweight deep neural network for surface scratch detection. Int. J. Adv. Manuf. Technol..

[26] Wang H., Chen K., Li Y. (2023). Automatic Detection Method for Black Smoke Vehicles Considering Motion Shadows. Sensors.

[27] Zheng J.C., Sun S.D., Zhao S.J. (2022). Fast ship detection based on lightweight YOLOv5 network. IET Image Process..

[28] Ioffe S., Szegedy C. (2015). Batch normalization: Accelerating deep network training by reducing internal covariate shift. Proceedings of the 32nd International Conference on Machine Learning (ICML 2015).

[29] Elfwing S., Uchibe E., Doya K. (2018). Sigmoid-weighted linear units for neural network function approximation in reinforcement learning. Neural Netw..

[30] Han K., Wang Y., Tian Q., Guo J., Xu C., Xu C. (2020). Ghostnet: More features from cheap operations. Proceedings of the 2020 IEEE/CVF Conference on Computer Vision and Pattern Recognition (CVPR).

[31] Tang Y., Han K., Guo J., Xu C., Xu C., Wang Y. (2022). GhostNetV2: Enhance Cheap Operation with Long-Range Attention. Adv. Neural Inf. Process. Syst..

[32] Hu J., Liu B., Peng S. (2019). Forecasting salinity time series using RF and ELM approaches coupled with decomposition techniques. Stoch. Environ. Res. Risk Assess..

[33] Sifre L., Mallat S. (2014). Rigid-motion scattering for texture classification. arXiv.

[34] Li H., Li J., Wei H., Liu Z., Zhan Z., Ren Q. (2022). Slim-neck by GSConv: A better design paradigm of detector architectures for autonomous vehicles. arXiv.

[35] Liu Y., Shao Z., Teng Y., Hoffmann N. (2021). NAM: Normalization-based attention module. arXiv.

[36] Woo S., Park J., Lee J.Y., Kweon I.S. (2018). Cbam: Convolutional block attention module. Proceedings of the 2018 European Conference on Computer Vision (ECCV).

[37] Rezatofighi H., Tsoi N., Gwak J., Sadeghian A., Reid I., Savarese S. (2019). Generalized intersection over union: A metric and a loss for bounding box regression. Proceedings of the 2019 IEEE/CVF Conference on Computer Vision and Pattern Recognition.

[38] Zheng Z., Wang P., Liu W., Li J., Ye R., Ren D. (2020). Distance-IoU loss: Faster and better learning for bounding box regression. Proceedings of the 2020 AAAI Conference on Artificial Intelligence.

[39] Gevorgyan Z. (2022). SIoU loss: More powerful learning for bounding box regression. arXiv.

